# Insights into venous thromboembolism prevention in hospitalized cancer patients: Lessons from a prospective study

**DOI:** 10.1371/journal.pone.0200220

**Published:** 2018-08-02

**Authors:** Rocío Figueroa, Ana Alfonso, José López-Picazo, Ignacio Gil-Bazo, Alberto García-Mouriz, José Hermida, José Antonio Páramo, Ramón Lecumberri

**Affiliations:** 1 Hematology Service, University Clinic of Navarra, Pamplona, Spain; 2 Oncology Department, University Clinic of Navarra, Pamplona, Spain; 3 Informatics Service, University Clinic of Navarra, Pamplona, Spain; 4 Centre for Applied Medical Research, University of Navarra, Pamplona, Spain; 5 Centro de Investigación Biomédica en Red (CIBER-CV), Instituto de Salud Carlos III, Madrid, Spain; Universite de Bretagne Occidentale, FRANCE

## Abstract

Hospitalized cancer patients are at high risk of venous thromboembolism (VTE). Despite current recommendations in clinical guidelines, thromboprophylaxis with low molecular weight heparin (LMWH) is underused. We performed an observational prospective study to analyse factors influencing prophylaxis use, VTE events and mortality in cancer-hospitalized patients. 1072 consecutive adult cancer patients were included in an University Hospital from April 2014 to February 2017, and followed-up for 30 days after discharge. The rate of LMWH prophylaxis was 67.6% (95% confidence interval [CI] 64.7% to 70.4%), with a 2.8% rate of VTE events (95% CI 1.9% to 3.9%) and 3.5% rate of major bleeding events (95% CI 2.5% to 4.8%). 80% of VTE events occurred despite appropriate thromboprophylaxis. Overall, 30-day mortality rate was 13.2% (95% CI 11.2% to 15.3%). Active chemotherapy treatment, hospital stay ≥ 4 days, and metastatic disease were associated with a higher use of LMWH. On the contrary, patients with hematologic malignancies, anemia or thrombocytopenia were less prone to receive thromboprophylaxis. The main reasons for not prescribing LMWH prophylaxis were thrombocytopenia (23.9%) and active/recent bleeding (21.8%). The PRETEMED score, used for VTE risk stratification, correlated with 30-day mortality. There is room for improvement in thromboprophylaxis use among hospitalized-cancer patients, especially among those with hematologic malignancies. A relevant number of VTE events occurred despite prophylaxis with LMWH. Therefore, identification of risk factors for thromboprophylaxis failure is needed.

## Introduction

Cancer is a well-known risk factor of venous thromboembolism (VTE). In fact, 20–30% of VTE events are related to an underlying malignancy [1, 2], sometimes being its first clinical manifestation [3, 4]. Outcome in terms of both recurrent thrombosis and bleeding is considerably worse in cancer-associated thrombosis (CAT), as well as the prognosis of the cancer itself in case of developing a VTE episode [5,6]. The morbidity and economic burden of CAT has encouraged different projects aimed to increase awareness on this health problem [7].

CAT is considered a potentially avoidable condition and several risk factors have been identified. Among them, hospital admission is one of the most frequent, contributing to approximately 50% of VTE events [8–10]. Despite specific studies in the cancer setting are lacking, clinical practice guidelines recommend routine prophylaxis with low molecular weight heparin (LMWH) in most hospitalized cancer patients, unless contraindicated, except for those admitted exclusively for chemotherapy infusion [11–12].

Data on pharmacologic thromboprophylaxis rate in hospitalized cancer patients are scarce, but uniformly show a global underuse. Retrospective studies have shown prophylaxis rates between 18%-56% [13–15]. A deeper knowledge of current thromboprophylaxis practice in hospitalized cancer patients and the factors that influence its use could be of value for health stakeholders in order to design appropriate strategies to improve quality of care. For this purpose, we have designed a prospective study, aimed to assess the rate of VTE pharmacologic prophylaxis use in cancer hospitalized patients in daily practice and identify variables related with its prescription and outcome.

## Materials and methods

### Study design

Prospective cohort study, including consecutive adult medical cancer patients (≥ 18 years-old), not receiving anticoagulant therapy, admitted in the Oncology and Hematology Departments at the University Clinic of Navarra from April 2014 to February 2017. The study was approved by the institutional Ethics Committee and all patients signed an informed consent.

The risk of VTE of all patients was automatically calculated daily by using an application of the Institution electronic clinical history software following the validated PRETEMED scale [16,17]. In this point scale major risk factors such as active cancer, previous VTE, acute myocardial infarction, ischaemic stroke with limb paralysis, decompensated chronic obstructive pulmonary disease and thrombophilia were assigned a score of 3; congestive heart failure, chronic renal insufficiency/nephrotic syndrome, severe acute infection, lower limb cast or prolonged bed rest were assigned a score of 2; pregnancy/postpartum period, recent prolonged flight, lower limb paresis, estrogen therapy, thalidomide/lenalidomide administration, use of central vein catheter, obesity, age > 60 years or smoking were assigned a score of 1. High risk of VTE was defined as a cumulative score of at least 4 points. According to our institutional thromboprophylaxis protocol, medical inpatients with high risk of VTE, including cancer patients, should receive 3500 IU of bemiparin daily, unless contraindicated. In case of withholding LMWH prophylaxis in a patient with high-risk score, the application asked the responsible physician to explain the reason.

Primary outcome of the study was the rate of thromboprophylaxis use. Secondary endpoints were the incidence of VTE, major bleeding and mortality during follow-up. VTE events had to be objectively confirmed by ultrasonography or contrast venography (for deep venous thrombosis [DVT]) and computed tomography or pulmonary ventilation/perfusion scan (for pulmonary embolism [PE]). Major bleeding was defined as any fatal bleeding and/or symptomatic bleeding in a critical area or organ, extra surgical site bleeding causing a fall in haemoglobin level ≥20 g/L, or leading to transfusion of two or more units of whole blood or red cells and/or surgical site bleeding that requires a second intervention or that is unexpected and prolonged and/ or sufficiently large to cause hemodynamic instability [18].

Follow-up period comprised from admission to 30 days after discharge. Main clinical data, VTE risk score and pharmacologic thromboprophylaxis use were provided by multiple sources of our institution electronic clinical history program. VTE, major bleeding or mortality after discharge were obtained either by outpatient visit or telephone interview.

### Statistical analysis

Categorical variables were expressed as frequencies and percentages, and quantitative variables as either mean (± standard deviation) or median depending on distribution. Correlation between clinical variables and outcomes was made by Pearson correlation coefficient (PCC) and multivariate analysis through regression logistics models. Odds ratios (OR) and its corresponding 95% confidence interval (95%CI) were calculated. Type I error was established in 0.05. Statistical analyses were performed using IBM SPSS Statistics (version 22.0, Chicago, IL, USA) software.

## Results

From April 2014 to February 2017, 1072 consecutive adult cancer patients were included. The main clinical features are shown in Table 1. Mean age was 62 ± 13 years and 58% were male. Overall, 20% of patients were diagnosed with a hematologic malignancy and 54.6% had advanced-stage cancer. At admission, 82% were receiving active chemotherapy treatment. Median hospital stay was 5 days (range: 1–140) and median PRETEMED score was 5 points (range: 3–13).

**Table 1:** Clinical characteristics of the patients.

	Total
**N**	1072
**Age (years ±SD** ^**a**^**)**	62.1 ± 13.3
**Sex (Male / Female)**	626/446
**Male (%)**	58.4%
**Median hospital stay (days) (Range)**	5 (1–140)
**Stay ≥ 4 days (n, %)**	662 (61.8%)
**Lung cancer (n, %)**	161 (15.01%)
**Colorectal cancer (n, %)**	177 (16.5%)
**Gastrointestinal cancer (n, %)**	96 (9%)
**Pancreas cancer (n, %)**	77 (7.2%)
**Breast cancer (n, %)**	42 (3.9%)
**Prostate cancer (n, %)**	37 (3.5%)
**Renal and urinary cancer (n, %)**	44 (4.1%)
**Gynecologic cancer (n, %)**	80 (7.5%)
**Hematologic cancer (n, %)**	217 (20.2%)
**Other cancer (n, %)**	141 (13.2%)
**Median PRETEMED score (range)**	5 (3–13)
**PRETEMED < 4 points (n, %)**	83 (7.7%)
**PRETEMED ≥ 4 points (n, %)**	989(2.3%)
**PRTEMED ≥ 7 points (n, %)**	294 (27.4%)
**Advanced stage (n, %)**	585 (54.6%)
**Active chemotherapy (n, %)**	880 (82.1%)
**Mean Platelets (x10**^**3**^**/L) (±SD** ^**a**^**)**	232 ± 129
**Platelets ≤ 50000/mm**^**3**^ **(n, %)**	63 (5.9%)
**Mean Leukocytes (x10**^**9**^**/L) (±SD** ^**a**^**)**	8.3 ± 7.6
**Mean Hemoglobin (g/dL) (±SD** ^**a**^**)**	11.2 ± 2.1
**Hemoglobin ≤10g/dL (n, %)**	277 (25.8%)

ª SD: Standard Deviation

The overall rate of thromboprophylaxis use during hospitalization was 67.6% (725/1072 patients) (95%CI 64.7% to 70.4%). Of note, prophylaxis was prescribed to 68% cancer inpatients with ≥4 points but also to 62% of those scoring less than 4 points. On multivariate analysis, active chemotherapy treatment (OR: 2.06, 95%CI, 1.3 to 3.10, p<0.001), hospital stay ≥ 4 days (OR: 2.36, 95%CI 1.5 to 3.4, p<0.001), and metastatic disease (OR: 1.6, 95%CI 0.9 to 2.5, p = 0.005) were associated with a higher use of LMWH. On the contrary, patients with hematologic malignancies (OR: 0.38, 95%CI 0.26 to 0.57, p<0.001), haemoglobin <10 g/dL (OR: 0.69, 95%CI 0.50 to 0.95, p = 0.026) or thrombocytopenia <50x10^9^/L (OR: 0.2, 95%CI 0.12 to 0.41, p<0.001), were less prone to receive thromboprophylaxis (Table 2). Main reasons for not prescribing LMWH prophylaxis according to responsible clinicians were thrombocytopenia (23.9%) and active/recent bleeding (21.8%). Remarkably, in 16.6% of cases the physician did not agree with the high VTE risk assigned by the software.

**Table 2:** Multivariate analysis of variables that influence thromboprophylaxis use in hospitalized cancer patients.

Variables	OR (CI 95%)	p
**Hospital stay ≥ 4 days**	2.36 (1.76–3.16)	< 0.001
**Advanced tumour stage**	1.60 (1.15–2.22)	0.005
**Active chemotherapy treatment**	2.06 (1.45–2.94)	< 0.001
**Platelets < 50 x10^9^/L**	0.2 (0.12–0.41)	< 0.001
**Haemoglobin ≤10g/dl**	0.69 (0.50–0.95)	0.026
**Hematologic tumour (vs Solid)**	0.38 (0.26–0.57)	< 0.001

During the follow-up period (hospitalization and 30 days after discharge), 30 VTE events, 3 of them incidental, were recorded (2.8%, 95%CI 1.9% to 3.9%); 8 lower limb DVT, 11 PE (+/- DVT), 10 central venous catheter-related thrombosis (CRT) and one mesenteric vein thrombosis. Nineteen events (63%) happened during admission and the rest after discharge, all of them in patients scoring ≥4 points. Of note, 24 of the VTE events (80%) developed despite appropriate thromboprophylaxis use during hospitalization.

Regarding major bleeding, 37 events were recorded (3.5%, 95%CI 2.5% to 4.8%). The most frequent location was gastrointestinal (n = 21; 56.7%), followed by skin and mucous membranes (n = 3; 8.1%), urinary system (n = 3; 8.1%) and intracranial (n = 2; 5.4%). Fourteen major bleeding events (37.8%) happened during admission and the rest after discharge. Thromboprophylaxis with LMWH did not increase the incidence of major bleeding (3.4% in patients with vs. 3.5% in patients without thromboprophylaxis).

Finally, 139 deaths were registered (13.2%, 95%CI 11.2% to 15.3%). The main cause of death was the malignancy itself (n = 101; 72.7%), followed by infections (n = 8; 5.7%). Four deaths (2.8%) happened as a result of arterial ischemic events (acute myocardial infarction or stroke), one death occurred because of PE and two deaths as a consequence of major bleeding (intracranial and gastrointestinal, respectively).

No significant correlation between any of the clinical variables and the risk of VTE or bleeding was observed. However, multivariate analysis showed that hospital stay ≥ 4 days (OR: 5.29, 95%CI 2.93 to 9.56, p<0.001), metastatic disease (OR: 4.44, 95%CI 2.75 to 7.15, p<0.001), PRETEMED score (OR: 1.67, 95%CI 1.11 to 2.48, p = 0.01), thrombocytopenia <50x10^9^/L (OR:3.49, 95%CI 1.75 to 6.99, p>0.0001) and major bleeding events (OR: 4.06, 95%CI 1.97 to 8.34, p<0.0001) were independently associated with an increased risk of 30-day mortality (Table 3). Mortality rate paralleled the PRETEMED score, increasing from 7.9% in patients scoring 3–4 points to 19.4% in those with ≥ 7 points. Thromboprophylaxis use was not associated with differences in mortality during follow-up.

**Table 3:** Multivariate analysis of variables that influence mortality.

Variables	OR (CI 95%)	p
**Hospital stay ≥ 4 days**	5.29 (2.93–9.56)	< 0.001
**Advanced tumour stage**	4.44 (2.75–7.15)	< 0.001
**PRETEMED Score risk ≥ 7 points**	1.67 (1.11–2.48)	0.01
**Platelets < 50 x10^9^/L**	3.49 (1.75–6.99)	< 0.001
**Major bleeding**	4.06 (1.97–8.34)	< 0.001

## Discussion

In this prospective study, we have evaluated the use of pharmacologic prophylaxis in cancer hospitalized patients in daily practice. The overall 67.6% rate is superior than that reported in previous retrospective studies, which ranged between 18%-56% [13–15] as well as in a previous assessment in our institution (57% in 2010) [19]. In a recent cross-sectional multicentre study in US, pharmacologic thromboprophylaxis was administered to 50.6% of cancer inpatients, although an additional 31.9% were considered to have relative contraindications [20]. Together with a growing general interest on CAT in the last years, the use of electronic alerts for VTE prevention, implemented at our centre since 2006 for all inpatients, may partially explain our positive results. No other intervention or campaign regarding VTE prevention has been conducted at our institution in the recent years. Our study did not consider possible contraindications for pharmacological prophylaxis, but physicians were asked about their reasons for not prescribing it at patients with high-risk of VTE. As expected, the main reasons were related with increased bleeding risk such as thrombocytopenia, recent bleeding or need of a high bleeding-risk procedure. However, in 16% of cases the clinician simply ignored the alert, implying that additional approaches may be required.

Our results confirm previous observation on predictive variables of thromboprophylaxis use in this population. Most of them seem logic from a clinical point of view, but others, such as hematologic malignancy are not supported by recent literature [20]. In fact, overall, patients with hematologic tumours show similar VTE rates than patients with solid malignancies (2.3% in our study). Further efforts aimed to improve thromboprophylaxis focused on this subgroup of patients are required. Of note, in our assessment the PRETEMED score was not a predictive factor for thromboprophylaxis use. The aforementioned cross-sectional study obtained similar findings applying the Padua score. However, in both scores the diagnosis of cancer is an important item and thus almost all patients were considered as high-risk.

Our VTE rate during or shortly after hospitalization (2.8%) is lower than others previously reported (4.1% in a multicentric retrospective study in 2007) [9]. On the other hand, in a recent prospective study in ambulatory cancer patients, the 90-day VTE rate was 5.2% [21]. The low rate of VTE could be at least partially explained by the current high rate of prophylaxis use at our centre. The fact that we did not observe higher incidence of major bleeding in patients receiving LMWH prophylaxis sustains the safety of this practice. Interestingly, 80% of the VTE events in our study occurred despite prophylaxis with LMWH had been administered. A Swiss registry described that up to 60% of VTE events in hospitalized cancer patients developed despite pharmacological prophylaxis [22]. Thus, the rate of prophylaxis failure with bemiparin, the LMWH used in our study, was 3.3% (24 events out of 725 patients), lower than the 8.9% dalteparin prophylaxis failure observed in the Artic study [23]. This difference, rather than different efficacy of both LMWH molecules, may be justified by the retrospective design of the Artic study. Indeed, identification of predictive variables of thromboprophylaxis failure are warranted. These patients could benefit of higher LMWH dose or longer duration of prophylaxis, provided their risk of bleeding is not high [24]. Unfortunately, due to the relatively low absolute number of VTE events, we were not able to shed light on this matter. In the Artic study, the only significant predictive factor of prophylaxis failure was repeated admissions. Moreover, in our study one third of the recorded VTE events were CRT. The effectiveness of LMWH prophylaxis for the prevention of catheter-related DVT is controversial. In fact, current guidelines do not recommend routine administration of LMWH for this purpose [25].

Regarding variables associated with mortality, longer hospital stay and advanced disease were strongly related in the multivariate analysis. Arterial or venous thrombotic complications were the third leading cause of mortality in our series, but by far the main cause was the malignancy itself. A correlation between the PRETEMED score and all-cause mortality in cancer patients was found. For the first time, this score is presented not only as a VTE risk assessment tool, but also as a potential predictor of 30 day-mortality in hospitalized cancer patients. Indeed, these results must be validated in future studies. Recently, another validated VTE clinical risk score, the Khorana score, has shown predictive value for early mortality and cancer progression in patients starting chemotherapy [26].

The present study has some limitations to acknowledge. As commented before, the total number of registered VTE and bleeding events is low, limiting the assessment of predictive risk factors. The probability of having missed any symptomatic or incidental VTE is minimal due to the prospective design of the study and that follow-up was performed by two investigators in charge of the management of all VTE episodes in our centre. Due to the design of the study, an independent adjudication committee was not established. Secondly, we lack reliable data on LMWH use after discharge. However, this is not common practice and only a minority of patients may have received extended out-of-hospital prophylaxis. Bemiparin, the LMWH used, is approved by regulatory agencies for both, prophylaxis (in surgical and non-surgical patients) and treatment of VTE. Albeit no large phase III clinical trials in medical inpatients have been conducted with this drug, there are several large phase III studies in the surgical setting (including a direct comparison with enoxaparin) as well as some prospective studies in medical patients [27]. Thirdly, this was a single-centre study. A multicentre study design would provide higher quality evidence, but our fully electronic clinical history system is not exportable, making hard an appropriate comparison. Finally, the impact of any particular antineoplastic therapy, for instance antiangiogenic drugs, was not evaluated. Therefore, some potential thrombotic or bleeding risk factor could have been missed.

In conclusion, more than two thirds of the cancer inpatients receive prophylaxis with LMWH. Nevertheless, future efforts to further improve thromboprophylaxis use are required, particularly for patients with hematologic malignancies. Although a low rate of VTE events was registered in our series, a relevant number occurred despite pharmacologic prophylaxis, therefore, identification of risk factors for thromboprophylaxis failure is needed. The PRETEMED score could be a potential tool to predict 30-day mortality in cancer hospitalized patients.
